# To Be or Not to Be an OXA-48 Carbapenemase

**DOI:** 10.3390/microorganisms10020258

**Published:** 2022-01-24

**Authors:** Laura Dabos, Saoussen Oueslati, Sandrine Bernabeu, Rémy A. Bonnin, Laurent Dortet, Thierry Naas

**Affiliations:** 1 Team “Resist” UMR1184 “Immunology of Viral, Auto-Immune, Hematological and Bacterial Diseases (IMVA-HB)”, INSERM, Université Paris-Saclay, CEA, LabEx LERMIT, Faculty of Medicine, 94270 Le Kremlin-Bicêtre, France; laura_dabos@hotmail.com (L.D.); Oueslati.saoussen@gmail.com (S.O.); Sandrine.bernabeu@aphp.fr (S.B.); remy.bonnin@u-psud.fr (R.A.B.); laurent.dortet@aphp.fr (L.D.); 2 Bacteriology-Hygiene Unit, Assistance Publique-Hôpitaux de Paris, AP-HP Paris-Saclay, Bicêtre Hospital, 94270 Le Kremlin-Bicêtre, France; 3 Associated French National Reference Center for Antibiotic Resistance: Carbapenemase-Producing Enterobacteriaceae, 94270 Le Kremlin-Bicêtre, France

**Keywords:** *k*_cat_/*K*_m_, coefficient of activity, carbapenem-hydrolysis, OXA-48-like, confirmatory tests

## Abstract

Since the first description of OXA-48, more than forty variants have been recovered from *Enterobacterales* isolates. Whereas some OXA-48-related enzymes have been reported as conferring similar resistance patterns, namely, the hydrolysis of carbapenems and penicillins with very weak or almost no activity against expanded-spectrum cephalosporins, some have reduced carbapenem and temocillin hydrolysis, and others hydrolyze expanded-spectrum cephalosporins and carbapenems only marginally. With such drastic differences in the hydrolytic profile, especially of carbapenems, it becomes urgent to establish hydrolytic cutoffs in order to determine when an OXA-48-like enzyme may be considered as a carbapenemase or not. With this aim, the coefficient of activity for imipenem (*k*_cat_/*K*_m_) was determined for a total of 30 enzymes, including OXA-48, OXA-48-like natural variants, and OXA-48 synthetic mutants. In addition, six different methods for the detection of carbapenemase-producers were performed. The coefficients of activity for imipenem for all the different enzymes went from 550 mM^−1^·s^−1^ to 0.02 mM^−1^·s^−1^. In order to match the coefficient of activity results with the biochemical confirmatory tests, we suggest the value of 0.27 mM^−1^·s^−1^ as the cutoff above which an OXA-48 variant may be considered a carbapenem-hydrolyzing enzyme.

## 1. Introduction

Carbapenem-hydrolyzing class D β-lactamases (CHDLs) have been most frequently found in *Acinetobacter* spp. [1,2,3], whereas OXA-48-type enzyme have been identified only in *Enterobacterales* [4,5,6,7,8]. Since its initial identification in a clinical *Klebsiella pneumoniae* isolate recovered in Istanbul, Turkey, in 2001 [4,5], an endemic spread of these bacteria has been reported in countries such as Turkey, Morocco, Libya, Egypt, Tunisia, and India [4,5,6,7,8]. In other countries (such as France, Spain, Italy, Belgium, the Netherlands, the UK, Germany, Switzerland, Lebanon, Israel, Kuwait, Saudi Arabia, China, and Japan), increasing descriptions are reported [4,5,6,7,8]. In many western European countries, high prevalence rates of OXA-48 carbapenemases among carbapenemase-producing *Enterobacterales* (CPEs) have been reported, best exemplified by Spain and France, with 74% and 70%, respectively [8,9].

Until today, more than 30 OXA-48-like variants have been reported from clinical Enterobacterales isolates worldwide [3,6,7,8]. For a complete list of variants, see the “Beta-Lactamase DataBase” [3], http://bldb.eu/BLDB.php?class=D#OXA, accessed on 13 January 2022 and the “Bacterial Antimicrobial Resistance Reference Gene Database” (https://www.ncbi.nlm.nih.gov/pathogens/isolates#/refgene/) (accessed on 13 January 2022). As displayed on the BLDB database, many more variants have been described in metagenomic data [3,10].

CHDLs of OXA-48-type are concerning, given their rapid worldwide spread and their propensity to evolve by mutations leading to various phenotypic expressions [11,12,13,14,15]. Although OXA-48 hydrolyzes penicillins at a high level, it hydrolyzes carbapenems only at a low level; however, OXA-48 is a class D β-lactamase with the highest known catalytic efficiency for imipenem (*k*_cat_ value of 5 s^−1^) [11]. In addition, it shows very weak activity against expanded-spectrum cephalosporins [11,14]. In fact, it hydrolyzes cefotaxime very poorly, but does not significantly hydrolyze ceftazidime and cefepime. Whereas some OXA-48-related enzymes such as OXA-181, and OXA-204 confer similar resistance pattern, e.g., hydrolysis of carbapenems and penicillins with very weak or almost no activity against expanded-spectrum cephalosporins, others have reduced carbapenem- and temocillin-hydrolysis, such as OXA-244 and OXA-232, and others such as OXA-163 and OXA-405 compromise the efficacy of broad-spectrum cephalosporins and hydrolyzes carbapenems only marginally [14,15,16]. These differences in carbapenem hydrolytic activity, especially with imipenem, raise the question as when to consider an OXA-48-variant as a carbapenemase, especially from a clinical perspective. The aim of this study is to determine a cutoff value that matches with the current diagnostic tests used for the confirmation of the presence of a carbapenemase and allow OXA-48-like enzymes to be classified as carbapenemase or not-carbapenemase.

## 2. Materials and Methods

### 2.1. Bacterial Strains

*Escherichia coli* TOP10 (Invitrogen, Saint-Aubin, France) was used for cloning experiments. *K. pneumoniae* 11978 was used as a donor strain for OXA-48 for subsequent mutagenesis and cloning experiments [4,5]. *E. coli* strain BL21 (DE3) was used for overexpression experiments.

### 2.2. Confirmatory Tests for Carbapenemase-Producing Enterobacterales

Carbapenemase activity was investigated using the in-house Carba NP test, RAPIDEC^®^ CARBA NP (BioMérieux, Paris, France), β-Carba^TM^ Test (BioRad, Marnes-la-Coquette, France), and CIM, rCIM and Maldi-TOF (MBT STAR-Carba^TM^, Brucker Daltonics, Wissenbourg, France). The tests were performed following the manufacturer’s recommendations or as previously published [17,18,19,20,21,22].

### 2.3. PCR, Cloning Experiments, and DNA Sequencing

The entire coding sequences of the β-lactamase genes (*bla*_OXA-48_, *bla*_OXA-162_, *bla*_OXA-163_, *bla*_OXA-181_, *bla*_OXA-204_, *bla*_OXA-232_, *bla*_OXA-405_, *bla*_OXA-517_, *bla*_OXA-519_, and *bla*_OXA-535_) [3] were obtained by PCR amplification using primers pre-OXA-48-Fw (5′ gcattaagcaaggggacgtt 3′) and preOXA-48-Rv (5′caaatacgcgctaaccactt 3′), as previously described [14]. Amplicons were then cloned into the pCR^®^-Blunt II-TOPO^®^ plasmid (Invitrogen, Illkirch, France) downstream of the pLac promoter and in the same orientation. pCR^®^-Blunt II-TOPO^®^ (Invitrogen, Saint-Aubin, France), harboring the *bla*_OXA-48_ gene, was used as a template for site-directed mutagenesis using a QuikChange II Site-Directed Mutagenesis Kit (Agilent Technologies, les Ulis, France), as previously described [13].

The mutated *bla*_OXA-48-like_ alleles corresponding to the mature OXA-48 β-lactamase were cloned into the expression vector pET41b (+) (Novagen, VWR International, Fontenay-sous-Bois, France) using the PCR-generated fragment with primers INF-OXA-48Fw (5′-AAGGAGATATACATATGGTAGCAAAGGAATGGCAAG-3′), INF-OXA-48Rv (5′-GGTGGTGGTGCTCGAAGGGAATA ATTTTTTCCTGTTTGAG-3′) and the NEBuilder^®^ HiFiDNA Assembly Cloning Kit (New England BioLabs, Évry-Courcouronnes, France), following the manufacturer’s instructions. Recombinant plasmids were transformed into chemocompetent *E. coli* strain BL21 (DE3).

Recombinant plasmids were extracted using the Qiagen miniprep kit and the inserts were sequenced on both strands with an automated sequencer (ABI Prism 3100, Applied Biosystems, Thermo Fischer Scientific, Villebon sur Yvette, France). The nucleotide sequences were analyzed using software available at the National Center for Biotechnology Information’s website (http://www.ncbi.nlm.nih.gov) (accessed on 13 January 2022).

### 2.4. β Lactamase Purification

Overnight cultures of *E. coli* strain BL21 (DE3) harboring the different pET41b-OXA-48/variant/mutated were used to inoculate 2 L of LB broth containing 50 µg/mL of kanamycin. Bacteria were cultured at 37 °C until reaching an OD of 0.6 at 600 nm. Expression of the different OXA-48/variant/mutations were induced overnight at 25 °C with 0.2 mM IPTG, as previously described [14]. OXA-48-like enzymes were purified in one-step pseudo-affinity chromatography using an NTA–nickel column (GE Healthcare, Les Ulis, France), as previously described [14]. Protein purity was estimated by SDS–PAGE, pure fractions were pooled and dialyzed against 20 mM Hepes, 50 mM K_2_SO_4_ buffer (pH 7.0) and concentrated by using Vivaspin^®^ columns (GE Healthcare, Les Ulis, France). Protein concentration was determined by Bradford Protein assay (Bio-Rad, Marnes-La-Coquette, France) [23].

### 2.5. Kinetic Studies

The kinetic parameters of the purified OXA-48, OXA-48 variants and OXA-48 mutants were determined at 30 °C in 100 mM sodium phosphate buffer (pH 7.0). The *k_ca_*_t_ and *K_m_* values were determined by analyzing hydrolysis of imipenem (Sigma–Aldrich, Saint-Quentin-Fallavier, France) under initial-rate conditions with an ULTROSPEC 2000 model UV spectrophotometer (GE Healthcare, Les Ulis, France) using the Eadie–Hofstee linearization of the Michaelis–Menten equation, as previously described [14,24].

## 3. Results

The coefficient of activity for imipenem for a total of 30 proteins comprising OXA-48 and OXA-48 natural variants and OXA-48 mutants (Table 1) were determined. The coefficient of activity for imipenem varied between 550 mM^−1^·s^−1^ and 0.02 mM^−1^·s^−1^ and corresponded to OXA-181 and OXA-ΔYSTRI, respectively (Table 1 [25]). The range in the k_cat_/K_m_ covered all the possible scenarios regarding the hydrolysis of imipenem, and how the different possibilities of action for OXA-48 variants with carbapenemase activity were implied, or not.

Regarding the biochemical confirmatory tests based on carbapenems hydrolysis, OXA-48, OXA-48-like and OXA-48-mutant *E. coli* TOP10 producers, expressing proteins with the coefficient of activity for imipenem between 550 mM^−1^·s^−1^ and 0.52 mM^−1^·s^−1^, were identified as carbapenemase producers by Carba NP test, RAPIDEC^®^ CARBA NP, β-Carba^TM^, CIM, rCIM and MBT STAR-Carba^TM^ tests. The isolates expressing proteins with a k_cat_/K_m_ between 0.39 mM^−1^·s^−1^ and 0.27 mM^−1^·s^−1^ were identified as carbapenemases producers by all the tests except for the home-made Carba NP test, whereas the commercially available assay (RAPIDEC^®^ CARBA NP) detected low carbapenem hydrolysis.

In order to match the coefficient of activity results with the biochemical confirmatory tests, we suggest the value of 0.27 mM^−1^·s^−1^ as the cutoff above which an OXA-48 variant may be considered a carbapenem-hydrolyzing enzyme. Using this 0.27 mM^−1^·s^−1^ cutoff value, most natural variants of OXA-48, such as OXA-181, OXA-162, OXA-204, OXA-232, OXA-244, OXA-438, OXA-517 and OXA-519 (Table 2), for which k_cat_/K_m_ values have been reported in the literature, are higher than 0.27 mM^−1^·s^−1^, and thus may be considered as carbapenemases, whereas OXA-163, OXA-405 and OXA-247 are clearly not carbapenemases (Table 2).

## 4. Discussion

*K_cat_*/*K_m_* values between 0.39 mM^−1^·s^−1^ and 0.27 mM^−1^·s^−1^ were identified as carbapenemases producers, except for in the Carba NP test. A sensitivity and specificity of 100% for carbapenemase-producing carbapenem-resistant Enterobacterales was initially reported for the Carba NP [30], but other studies have subsequently reported sensitivities <90%, in particular with OXA-48-like-producers, as these enzymes have weak carbapenemase activity compared with the carbapenemases [31,32,33]. Other studies reported even lower sensitivities ranging from 40% to 77% for the detection of an OXA-48-like enzyme producing Enterobacterales, with the Carba NP test [34].

All the isolates expressing proteins with a coefficient of activity lower than 0.27 mM^−1^·s^−1^ were reported as negative for the presence of carbapenemases by all the test methods; however, a discrepancy was observed in the results obtained with the β-CARBA^TM^ test. Four isolates were positive as carbapenemases, even though the k_cat_/K_m_ values of their proteins were between 0.14 mM^−1^·s^−1^ and 0.02 mM^−1^·s^−1^. It was previously reported that β-CARBA^TM^ detects carbapenemases as OXA-48 variants lacking significant carbapenemase activity, but which are still able to hydrolyze ceftazidime [19]. Although the exact chromogenic β-lactam compound present in the β-CARBA^TM^ test is not known, we could speculate that this molecule presents structural differences with imipenem.

Although OXA-48 presents one of the highest coefficients of activity for imipenem, natural OXA-48-variants display a large range of activity against imipenem. OXA-181 and OXA-162 have the highest *k_cat_*/*K_m_* value, whereas OXA-163 has the lowest [14,15,35]. This range of activity represents a major problem for identifying OXA-48-like producers in a routine setting, especially when only biochemical tests based on carbapenem-hydrolysis are used, such as Carba NP test, RAPIDEC^®^ CARBA NP, β-CARBA^TM^, CIM, rCIM and MBT STAR-Carba^TM^ tests. Indeed, isolates expressing proteins with a *k_cat_*/*K_m_* between 0.39 mM^−1^·s^−1^ and 0.27 mM^−1^·s^−1^ were identified as carbapenemases producers by all the tests except for the home-made Carba NP test, whereas the commercially available assay (RAPIDEC^®^ CARBA NP) detected the lowest carbapenem hydrolysis. In addition, the expression of the hydrolytic activity of an OXA-48 variant depends on the genetic support (chromosome, low and high copy number plasmid) and the intrinsic hydrolytic activity of the variant. This is best exemplified with OXA-48-variants presenting an R214G/S mutation in the ß5-B6 loop, such as OXA-244 [36,37]. Indeed, several groups have reported that the identification of OXA-244-producers remains a challenge for clinical microbiological laboratories due to the heterogeneity of the results obtained when biochemical tests are used [36,37], even though OXA-244 is clearly a carbapenemase (*k_cat_*/*K_m_* around 20 mM^−1^) but with its gene chromosome encoded. Thus, using purified enzymes, only the hydrolytic activities are measured, irrespectively of the genetic support and expression of the enzyme, and determine a minimum coefficient of activity for imipenem hydrolysis, which is still detectable by the most common biochemical confirmatory tests used.

## 5. Conclusions

According to our results, OXA-48-like enzymes may be considered as a carbapenemase only when the coefficient of activity for imipenem is 0.27 mM^−1^·s^−1^ or higher. Otherwise, an OXA-48 variant with a *k_cat_*/*K_m_* value below 0.27 mM^−1^·s^−1^ may be considered as a non-carbapenemase. Our results match biochemical tests performed in most routine laboratories for OXA-48 carbapenemase detection.

The question on whether carbapenems may be used to treat infections with isolates expressing OXA-48 proteins with a coefficient of activity lower than 0.27 mM^−1^·s^−1^ is still debatable. It is unlikely that these OXA-48-variants may revert to OXA-48, as they have undergone four AA deletions in the β5-β6 loop, but there could be compensatory mutations that may restore partial carbapenem-hydrolysis.

## Figures and Tables

**Table 1:** The coefficient of activity for imipenem hydrolysis of OXA-48, OXA-48 natural variants and OXA-48 mutants. The Carba NP test, RAPIDEC^®^ CARBA NP, β-Carba^TM^, CIM, rCIM and MBT STAR-Carba^TM^ test results of *E. coli* TOP10 expressing OXA-48, OXA-48 natural variants and OXA-48 mutants.

Mutant	*k*_cat_/*K*_m_ ^a^	β-Carba	Carba NP Test	RAPIDEC^®^ CARBA NP	MBT START-Carba	CIM	rCIM
^1^ OXA-181	550	+	+	+	H	+	+
^1^ OXA-162	420	+	+	+	H	+	+
^1^ OXA-204	420	+	+	+	H	+	+
^2^ OXA-48 ΔP	386	+	+	+	H	+	+
^3^ OXA-48	369	+	+	+	H	+	+
^2^ OXA-48 H90A	268	+	+	+	H	+	+
^2^ OXA-48 P217A	214	+	+	+	H	+	+
^2^ OXA-48 E185A-R186A	126	+	+	+	H	+	+
^2^ OXA-48 E185A-R186A-R189A	121	+	+	+	H	+	+
^2^ OXA-48 D229A	113	+	+	+	H	+	+
^2^ OXA-48 R189A	71	+	+	+	H	+	+
^4^ OXA-535	67	+	+	+	H	+	+
^2^ OXA-48 ΔEP	31	+	+	+	H	+	+
^1^ OXA-232	20	+	+	+	H	+	+
^2^ OXA-517	14	+	+	+	H	+	+
^2^ OXA-48 ΔIEP	6.2	+	+	+	H	+	+
^2^ OXA-48 ΔY	3.7	+	+	+	H	+	+
^5^ OXA-48Loop18	3.2	+	+	+	H	+	+
^6^ OXA-519	2.1	+	+	+	H	+	+
^2^ OXA-48 ΔYST	0.52	+	+	+	H	+	+
^2^ OXA-48 ΔYS	0.39	+	−	+	H	+	+
^2^ OXA-48 ΔRIEP	0.27	+	−	+	H	+	+
^2^ OXA-48 ΔYSTRIEP	0.21	−	−	−	NH	−	−
^7^ OXA-405	0.20	+	−	−	NH	−	−
^2^ OXA-48 ΔTRIEP	0.14	+	−	−	NH	−	−
^1^ OXA-163	0.06	+	−	−	NH	−	−
^2^ OXA-48 ΔYSTR	0.02	+	−	−	NH	−	−
^2^ OXA-48 ΔYSTRI	0.02	−	−	−	NH	−	−
^2^ OXA-48 ΔYSTRIE	0.02	−	−	−	NH	−	−
^2^ OXA-48 ΔSTRIEP	ND	−	−	−	NH	−	−

^a^ Values of *k*_cat_/*K*_m_ expressed in mM^−1^·s^−1^ of imipenem hydrolysis were from ^1^ Oueslati et al. [14]; ^2^ Dabos et al. [25]; ^3^ Docquier et al. [11]; ^4^ Dabos et al. [26]; ^5^ Dabos et al. [12]; ^6^ Dabos et al. [27]; ^7^ Oueslati et al. [15].

**Table 2:** Carbapenemase or non-carbapenemase of OXA-48 natural variants.

OXA-48 Variant	*k_cat_*/*K_M_* (mM^−1^·s^−1^) Imipenem	Phenotype
^1^ OXA-181	550	Carbapenemase
^1^ OXA-16	420	Carbapenemase
^1^ OXA-20	420	Carbapenemase
^2^ OXA-48	369	Carbapenemase
^3^ OXA-436	300	Carbapenemase
^1^ OXA-232	38	Carbapenemase
^4^ OXA-244	20	Carbapenemase
^5^ OXA-517	14	Carbapenemase
^6^ OXA-438	4.4	Carbapenemase
^7^ OXA-519	2.1	Carbapenemase
^8^ OXA-405	0.2	non-carbapenemase
^6^ OXA-247	0.2	non-carbapenemase
^1^ OXA-163	0.06	non-carbapenemase

Values of *k*_cat_/*K*_m_ from ^1^ Oueslati et al. [14]; ^2^ Docquier et al. [11]; ^3^ Lund et al. [28]; ^4^ Rima et al. [16]; ^5^ Dabos et al. [25]; ^6^ De Belder et al. [29]; ^7^ Dabos et al. [27]; ^8^ Oueslati et al. [14].
